# Existing joint erosions increase the risk of joint space narrowing independently of clinical synovitis in patients with early rheumatoid arthritis

**DOI:** 10.1186/s13075-015-0626-1

**Published:** 2015-05-21

**Authors:** Robert Landewé, Josef S Smolen, Stefan Florentinus, Su Chen, Benoît Guérette, Désirée van der Heijde

**Affiliations:** Amsterdam Rheumatology Center, Amsterdam, The Netherlands and Atrium Medical Center, Heerlen, The Netherlands; Medical University of Vienna, Vienna, Austria; AbbVie, Rungis, France; AbbVie Inc., North Chicago, IL USA; Leiden University Medical Center, Leiden, The Netherlands

## Abstract

**Introduction:**

Clinical synovitis is often associated with damage to bone and cartilage. Previous data have suggested that joint erosions (JE) are more prevalent than joint space narrowing (JSN) and that the two processes are partly independent of each other. The objective of this study was to evaluate whether the presence of JE in an individual joint can lead to development of JSN and if existing JSN leads to new onset of JE, in the absence of synovitis.

**Methods:**

The Prospective Multi-Centre Randomised, Double-Blind, Active Comparator-Controlled, Parallel-Groups Study Comparing the Fully Human Monoclonal Anti-TNFα Antibody Adalimumab Given Every Second Week With Methotrexate Given Weekly and the Combination of Adalimumab and Methotrexate Administered Over 2 Years in Patients With Early Rheumatoid Arthritis (PREMIER) enrolled early rheumatoid arthritis (RA) patients who were randomized to one of three treatments: methotrexate (MTX), adalimumab (ADA), or ADA + MTX. All evaluable joints with JE and JSN measures at 26 and 52 weeks and synovitis assessments from week 26 to 52 were included. Synovitis was assessed every 2–8 weeks by swollen joint counts between weeks 26 and 52. Radiographs were taken at week 26 and 52. Two readers, blinded to time and sequence, scored 14 bilateral joints individually for JE and JSN. Multivariate logistic modeling was used to characterize the dependence of JE/JSN onset at 52 weeks. Analyses were performed based on treatment arm and were also performed within individual joints.

**Results:**

JE and swelling were independently and comparably associated with onset of JSN at week 52. Assessment by individual joints indicated that existing JE, independent of swelling, was significantly associated with JSN onset in higher proportions of metatarsophalangeal (MTP; 7/10) than proximal interphalangeal (PIP; 1/8) or metacarpophalangeal (MCP; 1/10) joints. Treatment with ADA + MTX prevents JE/JSN progression independently of its ability to suppress synovitis and limits JE/JSN onset and progression in joints with existing damage.

**Conclusions:**

Existing JE predisposes individual joints to development of JSN independently of synovitis in the same joint. Weight-bearing MTP joints with JE may be at increased risk for JSN when compared with MCPs and PIPs.

**Trial registration:**

Clinicaltrials.gov NCT00195663. Registered 13 September 2005.

**Electronic supplementary material:**

The online version of this article (doi:10.1186/s13075-015-0626-1) contains supplementary material, which is available to authorized users.

## Introduction

Rheumatoid arthritis (RA) is characterized by inflammation of the synovial membrane, which can result in joint destruction within the first few years after onset [1-3]. Inflammation and joint damage can result in a loss of physical function, which is a hallmark of progressive disease [4-7]. Damage to the bone is measured through joint erosion (JE), while cartilage injury is approximated by measuring joint space narrowing (JSN). Although JSN is likely a marker for loss of cartilage, it may also reflect damage to other soft tissues. JE has historically been perceived to be a critical indicator of permanent disability in RA patients; however, recent data suggest that JSN, occurring early in disease process, may be a more important determinant of irreversible physical disability [8,9].

The combination of a tumor necrosis factor (TNF) inhibitor plus methotrexate (MTX) reduces the risk of joint damage onset or progression and improves physical function more effectively than MTX monotherapy [10-15]. Patients treated with combination therapy tend to have minimal or no progression of joint damage, regardless of the level of inflammation, while the level of joint damage tends to reflect the extent of inflammation in MTX monotherapy [16-20]. This suggests that combination therapy may inhibit joint damage through mechanisms that are independent of inflammatory activity or that the extent of the biological inflammatory response required for the activation of destructive mechanisms may be higher than is needed for inducing the clinical signs and symptoms of inflammation [21].

Previous data have suggested that JE is more prevalent than JSN in early RA, as well as in advanced RA, and that the two processes are partly independent of each other [22]. It remains to be elucidated whether an individual joint with existing JSN and no signs of clinical synovitis is predisposed to future development of JE. Similarly, the relationship between existing JE and new onset of JSN needs further consideration***.*** In the present analysis, we evaluated the effects of three different therapies, MTX monotherapy, adalimumab (ADA) monotherapy, and ADA + MTX, on the relationship between existing JSN, clinical synovitis, and the predisposition of future JE development and, similarly, existing JE, clinical synovitis, and the predisposition of future JSN development in individual joints using data from a randomized, controlled trial in a population of patients with early RA.

## Methods

### Study design and patients

Data from patients in the Prospective Multi-centre Randomised, Double-Blind, Active Comparator-Controlled, Parallel-Groups Study Comparing the Fully Human Monoclonal Anti-TNFα Antibody Adalimumab Given Every Second Week With Methotrexate Given Weekly and the Combination of Adalimumab and Methotrexate Administered Over 2 Years in Patients With Early Rheumatoid Arthritis (PREMIER; NCT00195663) were used for all analyses [14]. PREMIER was a 104-week, phase 3, randomized, placebo-controlled trial of ADA in an MTX-naïve population with early RA. Adult patients (≥18 years of age) diagnosed with RA by the 1987 revised American College of Rheumatology criteria [23], with disease of <3 years duration, were eligible for enrolment if they satisfied entry criteria, as previously described [14]. All patients provided written, informed consent, and the study protocols and informed consent forms were approved by the local institutional review boards or independent ethics committees at participating sites (see Additional file 1). The study was conducted in accordance with the principles of the Declaration of Helsinki and good clinical practice.

### Radiographic, clinical, and functional assessments

Data for all evaluable joints with JE and JSN measures at 26 and 52 weeks and clinical synovitis assessments from weeks 26 to 52 were included in this post hoc analysis. Week 26 was chosen for the baseline assessment of JE, JSN, and synovitis because all patients had active disease at week 0; therefore, it is likely that many of the joints assessed would have shown clinical synovitis at the earlier time. Using week 26 as the new baseline facilitated evaluation of the relationship between JSN and JE, independent of clinical synovitis, since a greater proportion of patients would be expected to be free of swelling after 26 weeks of treatment.

Recent onset of JE/JSN was defined as the lack of JE/JSN at week 26 but JE/JSN presence at week 52. In contrast, existing JE/JSN was defined as the presence of JE/JSN at week 26. Clinical synovitis was assessed through swollen joint counts at specified visits occurring between weeks 26 and 52 (every 2 to 8 weeks). Ever swelling was defined as the presence of swelling within the individual joint at any visit from weeks 26 through 52. Radiographs of the hands (posteroanterior view) and feet (anteroposterior view) were taken at week 26 and week 52. Two readers, blinded to patient and sequence, evaluated joints (yes/no) individually for bone erosion (JE) and cartilage destruction (JSN), and only the subset of joints that were evaluated for JE and JSN and clinical synovitis were considered in the present analysis. The average score from the two readers was used for JE/JSN, and the presence of JE/JSN was defined as the average score >0 (operationalized as a dichotomous variable: a score of 0 versus a score >0). In the present analysis, only scores for the metacarpophalangeal (MCP) joints 1 to 5, proximal interphalangeal (PIP) joints 2 to 5, and metatarsophalangeal (MTP) joints 1 to 5 (total of 14 joints bilaterally) were assessed. Joints with recent onset of JE/JSN at week 52 were separated based on the presence or absence (dichotomous variable) of JSN/JE at week 26, respectively.

### Statistical analysis

Multivariate logistic modeling was used to characterize the dependence of JE/JSN onset at 52 weeks, which was selected because of the quantity of data available, on the following independent variables: ever swelling during the evaluable time (yes versus no), JE/JSN presence at 26 weeks (yes versus no), treatment, joint type (MCP, PIP, MTP), and body position (left, right). Alternatively, continuous JE/JSN scores, instead of the dichotomous variable, JE/JSN presence at week 26, were used in a multivariate logistic model to evaluate consistency in the models. In addition, analyses were also performed within each treatment arm (treatment no longer an independent variable) and for each individual joint (joint type and body position no longer independent variables).

## Results

### Baseline demographics and disease characteristics

This post hoc analysis evaluated joint-level data from 631 of the 799 patients randomized to MTX monotherapy (n = 202), ADA monotherapy (n = 203), or ADA + MTX combination therapy (n = 226) in PREMIER who had radiographs available at weeks 26 and 52 [14]. Both baseline demographics and disease characteristics were comparable among the three treatment groups (Table 1). The mean RA duration at baseline was 0.7 to 0.8 years, and there were similar percentages of patients who were rheumatoid factor-positive among each of the treatment arms. Approximately one third of the patients in each group had previously taken disease-modifying antirheumatic drugs (DMARDs) and used corticosteroids at baseline. The patients overall had a baseline 28-joint disease activity score based on C-reactive protein (DAS28-CRP) of 6.3 ± 0.9, and reported severe disability (disability index of the health assessment questionnaire) in each of the treatment arms. Mean baseline modified total Sharp score (mTSS), JE, and JSN scores were higher in the MTX monotherapy treatment group than in the ADA monotherapy or ADA + MTX treatment groups. Patient disease characteristics at baseline were comparable with those of the overall PREMIER population and were indicative of a population with early and severely active RA.

**Table 1:** **Baseline demographic and disease characteristics**

**Baseline characteristic**	**MTX**	**ADA**	**ADA + MTX**
	**(n = 202)**	**(n = 203)**	**(n = 226)**
Age, years	52.7 ± 13.3	52.0 ± 12.9	52.0 ± 14.1
Female, n (%)	149 (73.8)	154 (75.9)	159 (70.4)
Disease duration, years	0.8 ± 0.9	0.7 ± 0.8	0.7 ± 0.8
Rheumatoid factor-positive, n (%)	172 (85.6)	172 (84.7)	191 (84.5)
Prior DMARD use, n (%)	61 (30.2)	68 (33.5)	69 (30.5)
Baseline corticosteroid use, n (%)	68 (33.7)	74 (36.5)	81 (35.8)
SJC (0 to 66)	22.3 ± 12.0	21.6 ± 10.4	21.4 ± 11.5
TJC (0 to 68)	32.0 ± 14.3	32.5 ± 13.8	30.5 ± 14.5
DAS28-CRP (0 to 10)	6.3 ± 0.9	6.3 ± 0.9	6.3 ± 0.9
DAS28-CRP ≥5.1, n (%)	183 (93.4)	177 (88.9)	196 (90.3)
HAQ-DI (0 to 3)	1.5 ± 0.7	1.6 ± 0.6	1.5 ± 0.6
CRP, mg/dL	4.0 ± 4.0	3.8 ± 3.6	3.9 ± 4.1
mTSS (0 to 398)	22.5 ± 22.6	19.0 ± 18.9	18.7 ± 20.7
JE (0 to 230)^a^	14.0 ± 13.7	11.5 ± 11.5	11.4 ± 12.9
JSN (0 to 168)	8.5 ± 10.9	7.5 ± 8.8	7.3 ± 9.4

All values are mean ± SD, unless otherwise indicated. ^a^ *P* = 0.047 for pairwise comparison of MTX versus ADA + MTX. MTX, methotrexate; ADA, adalimumab; DMARD, disease-modifying antirheumatic drug; SJC, swollen joint count; TJC, tender joint count; DAS28, 28-joint disease activity score; HAQ-DI, disability index of the health assessment questionnaire; CRP, C-reactive protein; mTSS, modified total Sharp score; JE, joint erosion; JSN, joint space narrowing.

Of the 17,644 evaluable joints, 275 joints (1.6%), from 189 patients, lacked JE at week 26 but had evidence of JE by week 52 (Table 2). In addition, 226 (1.3%) joints, from 153 patients, lacked JSN at week 26 but had evidence of JSN by week 52. A total of 59 out of 275 joints (21.5%) with recent onset JE had existing JSN at week 26; in contrast, the majority of joints (133/226, 58.8%) with recent onset of JSN had existing JE at week 26. Joints with existing JE had a larger number of affected joints with recent onset JSN at week 52 than existing JSN with recent onset JE, which suggests that existing JE may have a greater impact on JSN appearance than existing JSN has on the appearance of JE in patients with early RA. There were 626 (99.2%) patients with at least one unaffected joint, as they did not have JE at week 26 or 52 nor JSN at week 26 or 52. Furthermore, 9,626 joints had no presence of JE or JSN, and, on average, 15 (of the 28 assessed) joints per patient were never affected by JE or JSN (data not shown).

**Table 2:** **Numbers of patients and joints with recent onset>** ^**a**^ **joint erosion** **(JE)/joint space narrowing (JSN) at 52 weeks exhibiting JSN/JE** **at week 26** ^**b**^

**Variable**	**Patients**	**Joints**
**n (%)**	**(n = 631** ^**c**^ **)**	**(n = 17,644)**
Recent onset JE with existing JSN	46 (7.3)	59 (0.3)
Recent onset JE without existing JSN	143 (22.7)	216 (1.2)
Recent onset JSN with existing JE	88 (13.9)	133 (0.8)
Recent onset JSN without existing JE	65 (10.3)	93 (0.5)

^a^No JE/JSN at week 26 but JE/JSN present at week 52. ^b^Existing JE/JSN is defined as the presence of JE/JSN at week 26. ^c^Patients could have different JE/JSN patterns for the joints that were evaluated.

### Association between existing JE, clinical synovitis, and treatment, and JSN onset in joints overall

**Figure 1:**
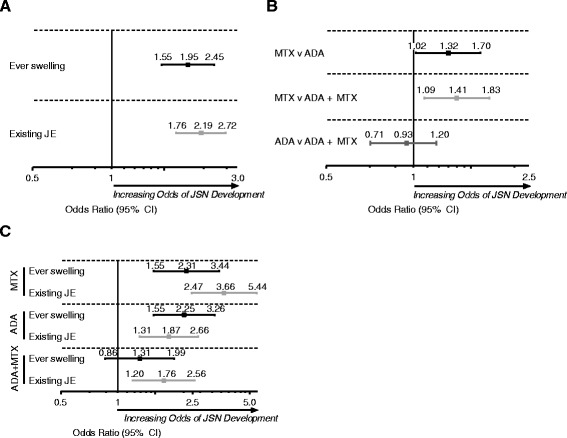
Association between existing JE, clinical synovitis, and treatment, and JSN onset in joints overall. **(A)** Ever swelling from 26 through 52 weeks and joint erosion (JE) presence at week 26 were independent predictors of presence of joint space narrowing (JSN) at 52 weeks. **(B)** The effect of treatment on the development of JSN at 52 weeks in joints with similar characteristics. **(C)** The association between ever swelling from 26 through 52 weeks and presence of JE at week 26, and JSN presence at 52 weeks, by treatment group. MTX, methotrexate; ADA, adalimumab*.*

In the multivariate logistic analyses, ever swelling from weeks 26 through 52 (odds ratio (95% CI), 1.95 (1.55, 2.45)) and existing JE at week 26 (2.19 (1.76, 2.72)) were significant predictors of JSN onset at week 52 (Figure 1A). The result from using continuous JE scores at week 26 validated the primary results, where higher JE scores at week 26 were associated with an increased risk of JSN onset by week 52 (1.80 (1.53, 2.10)). Treatment with ADA, either as monotherapy or in combination with MTX, reduced the risk of JSN development at week 52 for joints when controlling for other characteristics (for example, ever swelling, existing JE, et cetera) compared with MTX (Figure 1B). Ever swelling during the assessment period was a predictor of JSN recent onset in individual joints of patients treated with MTX (2.31 (1.55, 3.44)) and ADA (2.25 (1.55, 3.26)) monotherapy, but to a lesser, though not significant, extent of patients treated with the combination (1.31 (0.86, 1.99)) (Figure 1C), similar to the observed association between existing JSN and recent JE onset. Irrespective of treatment group, the presence of JE at week 26 was a significant predictor of JSN presence at week 52.

### The association between existing JSN, clinical synovitis, and treatment, and JE onset in joints overall

**Figure 2:**
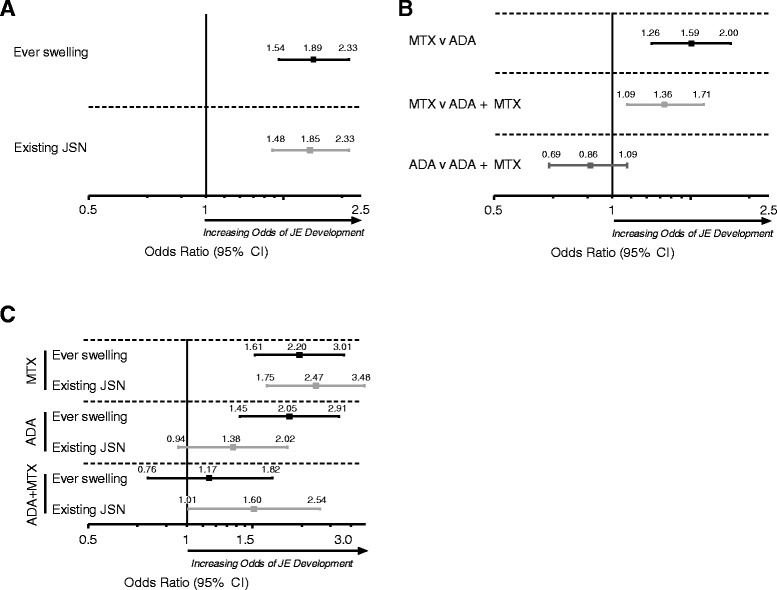
Association between existing JSN, clinical synovitis, and treatment, and JE onset in joints overall. **(A)** Ever swelling from 26 through 52 weeks and presence of joint space narrowing (JSN) at week 26 were independent predictors of presence of joint erosion (JE) at 52 weeks. **(B)** Effect of treatment on the development of JE at 52 weeks in joints with similar characteristics. **(C)** Association between ever swelling from 26 through 52 weeks and presence of JSN at week 26, and presence of JE at 52 weeks, by treatment group. MTX, methotrexate; ADA, adalimumab.

In the multivariate logistic analyses, ever swelling from weeks 26 through 52 and also existing JSN at week 26 were significant predictors of JE onset at week 52 (Figure 2A) (1.89 (1.54, 2.33) and 1.85 (1.48, 2.33), respectively). The result from using continuous JSN scores at week 26 validated these results, where higher JSN scores, indicative of more extensive cartilage damage, at week 26 were also associated with an increased risk of JE onset by week 52 (1.36 (1.14, 1.63)). Treatment with ADA, either as monotherapy or in combination with MTX, reduced the risk of JE development at week 52 for joints when controlling for other characteristics (for example, ever swelling, existing JSN, et cetera) compared with MTX (Figure 2B). During the treatment period, ever swelling was a significant predictor of JE recent onset in individual joints of patients treated with both MTX (2.20 (1.61, 3.01)) and ADA (2.05 (1.45, 2.91)) monotherapy (Figure 2C), but not of patients treated with the ADA + MTX combination (1.17 (0.76, 1.82)). These findings confirm the previous observation that ADA + MTX controls bone damage independently of its ability to control for disease activity [24]. Existing JSN at week 26 was also a predictor of JE onset in patients treated with MTX monotherapy (2.47 (1.75, 3.48)) or ADA + MTX combination therapy (1.60 (1.01, 2.54)); however, existing JSN did not significantly predict JE onset in patients who received ADA monotherapy (1.38 (0.94, 2.02)).

### The association between existing JE and clinical synovitis, and JSN onset in individual joints

**Figure 3:**
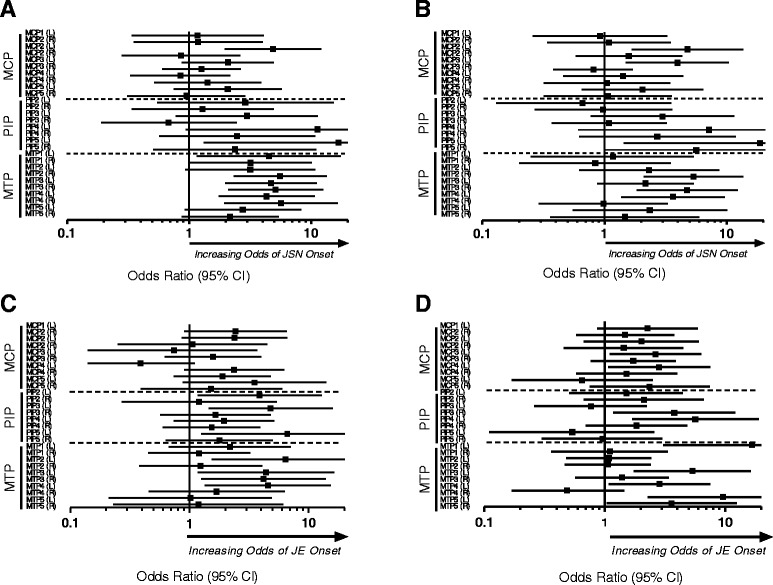
Association between existing JE, clinical synovitis, and JSN, and JSN and JE onset in individual joints. **(A)** Odds ratios (95% CI) for the influence of existing joint erosions (JE) to predict future onset of joint space narrowing (JSN) in individual joint assessments. **(B)** Odds ratios (95% CI) for the influence of joint swelling to predict future onset of JSN in individual joint assessments. **(C)** Odds ratios (95% CI) for the influence of existing JSN to predict future onset of JE in individual joint assessments. **(D)** Odds ratios (95% CI) for the influence of joint swelling to predict future onset of JE in individual joint assessments. MCP, metacarpophalangeal; L, left; R, right; PIP, proximal interphalangeal; MTP, metatarsophalangeal.

Individual joint assessments indicated that existing JE, independent of swelling, was a significant predictor of JSN onset in higher proportions of MTPs (7/10) than PIPs (1/8) or MCPs (1/10) (Figure 3A). Clinical synovitis was significantly associated with the onset of JSN in higher proportions of MTPs (3/10) than MCPs (2/10) or PIPs (1/8) (Figure 3B); however, the latter associations occurred in lower proportions than were observed with existing JE.

## Discussion

Joint damage is a hallmark of RA, and previous data have suggested that the processes of JE and JSN are partly independent of each other [22]. The objective of this study was to evaluate whether the presence of JE in an individual joint can lead to the development of JSN and if existing JSN leads to new onset of JE, in the absence of synovitis. Overall, our results suggest that existing JE may have a great impact on JSN development, and even more so than existing JSN has on subsequent JE development. Treatment with the combination of ADA + MTX prevents JE and JSN progression independently of its ability to suppress clinical synovitis and, furthermore, limits JE and JSN onset/progression in joints with existing damage. Additionally, weight-bearing joints, such as MTP joints, with JE may be at increased risk for JSN when compared with MCPs and PIPs.

The majority of previous analyses focused on combining all joints together, and, therefore, the impact of therapy, irrespective of inhibition of disease activity, was highly biased by the fact that synovitis may have not been observed in the very same joints than those that showed radiographic progression. Similar to the TEMPO trial [25], our study isolated each joint and recorded whether or not they had synovitis and/or onset of JE or JSN. Thus, at a joint level, we have shown that ADA + MTX prevents JE and JSN by effects beyond its ability to control for synovitis.

This analysis expands upon previous findings by suggesting that existing JE may predispose joints to further develop JSN, irrespective of the presence or absence of clinical synovitis. In addition, the independent impact of existing JSN on new onset of JE was also demonstrated in this study. Additionally, these findings suggest that joint erosions are more prevalent than JSN and occur earlier in the disease. However, this may also be a consequence of discrepancy in the scoring techniques related to JE and JSN, which may have distinct sensitivities to change. This post hoc analysis demonstrated that over 50% of the evaluable joints that had existing JE at week 26 developed recent onset of JSN, while only 21.5% of those joints with existing JSN at week 26 developed onset of JE. In addition, ever swelling and existing JSN at week 26 were significant predictors of JE onset at week 52, while ever swelling and existing JE at week 26 were significant predictors of JSN onset at week 52. Higher JSN or JE scores at week 26 were associated with an increased risk of JE or JSN onset by week 52, respectively, and these results further enhance the idea that the two processes are intertwined. In line with previous findings, our study also showed that treatment with ADA, either as monotherapy or in combination with MTX, reduced the risk of both JE and JSN development at week 52 for joints with similar characteristics when compared with MTX monotherapy.

Additionally, it has been shown here that weight-bearing MTP joints with JE may be at an increased risk of JSN when compared with MCPs and PIPs. Studies by Goldring *et al*. [26] suggest that chondrocytes can be activated by both pressure and mechanical stress, and are capable of stimulating signaling pathways that cause cartilage damage and defective repair mechanisms. Thus, a change in the geometric orientation of the joints, subsequent to significant JE, may result in increased pressure on the cartilage and activation of the chondrocytes, leading to JSN, which may be more prevalent in weight-bearing joints as they may be more sensitive to this mechanism of action.

A final question arises: can we possibly explain why joint erosions are a risk factor for development or progression of cartilage damage even in the absence of synovitis? The answer can only be speculative, but may give rise to further considerations. First, as discussed above, there may be mechanical changes due to the presence of erosions with increased cartilage wear. Second, a joint should only be diagnosed as swollen if the swelling is beyond doubt [27]. Thus, synovitis may exist in some joints without being clinically overt [28], and the presence of power Doppler signals of 3, in the absence of clinical joint swelling among RA patients, is associated with decreased physical function [29]. Third, RA patients have higher articular TNF levels than patients with less destructive forms of arthritis [30] and osteoclastogenesis requires higher amounts of TNF than synovial inflammation [21]; thus, incomplete TNF inhibition will reduce bioactive TNF levels below the threshold of osteoclastogenesis stimulation, even if leaving sufficient TNF to continue eliciting inflammation [31]. Therefore, it may be assumed that, even in subclinical synovitis, sufficiently high TNF concentrations arise that may elicit cartilage damage. Fourth, in a TNF-driven experimental model of RA, it has been seen that severe cartilage damage requires attachment of the synovial membrane to the cartilage surface [32]; aside from subclinical disease, attachment may persist for some time even when overall synovitic activity diminishes. Finally, bone erosions in RA frequently occur at the cartilage bone junction with synovial tissue (pannus) undermining cartilage and, thus, attacking cartilage in a forceps-like manner from above and below. Even if synovitis fades away, such double-edged activity may persist for some time, allowing cartilage damage to accumulate. As stated above, whether any one or all of these ideas play causative roles in our observation remains elusive, but they may provide hypotheses that lend themselves to further investigations in experimental settings.

The finding that progression of JE is absent while progression of JSN continues indicates that the two processes can be uncoupled. This concept is supported by data from an experimental model [21] that demonstrated that TNF blockade can interfere with osteoclastogenesis and expression of osteoclast-related genes, such as cathepsin K, and thus inhibit the erosive process while simultaneously leaving IL-1 gene expression fully intact. Because IL-1 is a major driver of cartilage damage, low IL-1 levels protect against cartilage damage even in an environment of high TNF levels [32]. In other words, cartilage damage is dependent on TNF and IL-1 in the joints, causing synovitis, whereas the development of erosions is dependent on the formation of osteoclasts, which requires more TNF than does synovitis. Conditions in our study were TNF inhibiting we can speculate that the TNF inhibition may have been sufficient to suppress osteoclastogenesis, but insufficient to prevent synovitis. Clinically, this would translate to a joint with swelling and progression of JSN, but without progression of erosions.

One of the limitations of our study is that JSN is not a direct measure of cartilage damage. The contribution of damage from the surrounding tissues is less likely to occur in patients with early RA; however, although our results should be validated in other RA populations, PREMIER was a study of patients with early RA and it is unlikely that major soft tissue changes occurred within the one-year period related to this analysis, especially as all patients received active medication.

## Conclusions

In summary, our results suggest that existing JE or JSN not only leads to more JE or JSN, but, at the joint level, existing JE may also lead to JSN onset in joints with no clinical synovitis. Similarly, existing JSN may also lead to JE onset in joints with no clinical synovitis. Both the combination of ADA + MTX and ADA monotherapy were associated with lower risk of JE or JSN onset than was MTX monotherapy in individual joints with similar characteristics, irrespective of the presence of synovitis. Given the impact of JSN on physical function early in the disease course, this further highlights the need for effective therapies that can rapidly control for synovitis and inhibit the onset of both JE and JSN.

## Additional file

Additional file 1:
**List of investigator sites and corresponding ethics committees/review boards.**

## Abbreviations

ADA: adalimumab
CRP: C-reactive protein
DAS28: 28-joint disease activity score
DMARDs: disease-modifying antirheumatic drugs
HAQ-DI: disability index of the health assessment questionnaire
JE: joint erosion
JSN: joint space narrowing
MCP: metacarpophalangeal
MTP: metatarsophalangeal
mTSS: modified total Sharp score
MTX: methotrexate
PIP: proximal interphalangeal
PREMIER: Prospective Multi-Centre Randomised, Double-Blind, Active Comparator-Controlled, Parallel-Groups Study Comparing the Fully Human Monoclonal Anti-TNFα Antibody Adalimumab Given Every Second Week With Methotrexate Given Weekly and the Combination of Adalimumab and Methotrexate Administered Over 2 Years in Patients With Early Rheumatoid Arthritis
RA: rheumatoid arthritis
SJC: swollen joint count
TJC: tender joint count
TNF: tumor necrosis factor
