# Supplementary material for: Existing joint erosions increase the risk of joint space narrowing independently of clinical synovitis in patients with early rheumatoid arthritis
Source: Arthritis Res Ther. 2015 May 21;17(1):133. doi: 10.1186/s13075-015-0626-1 (PMC4440309; doi:10.1186/s13075-015-0626-1)
Supplement: Additional file 1: — List of investigator sites and corresponding ethics committees/review boards. [file 13075_2015_626_MOESM1_ESM.pdf]

List of Investigators and Corresponding IEC/IRB			
	**Site Name/Post Office Address**	**Name and Address of IEC/IRB**	
	Azienda Ospedaliera Universitaria Integrata Verona<br>Policlinico G.B. Rossi, Instituto di Patologia Speciale Medica Cattedra di Reumatologia P. le L.A. Scuro, 10 Verona 37134 Italy	Comitato Etico per la sperimentazione dell'Azienda Ospedaliera Universitaria Integrata Verona -P.le Stefani, 1 37126 Verona Italy	
	The Center for Rheumatology and Bone Research 2730 University Boulevard West Suite 306 Wheaton, MD 20902 United States	Western International Review Board 3535 Seventh Avenue SW Olympia, WA 98502 United States	
	Portland Rheumatology Clinic, LLC<br>17050 Pilkington Road, Suite 130<br>Lake Oswego, OR 97035 United States	Western International Review Board<br>3535 Seventh Avenue SW<br>Olympia, WA 98502 United States	

List of Investigators and Corresponding IEC/IRB			
	**Site Name/Post Office Address**	**Name and Address of IEC/IRB**	
	Sunnybrook Health Sciences Centre 2075 Bayview Avenue Room M1-430 Toronto, ON M4N 3M5 Canada	Sunnybrook Health Sciences Centre Research Ethics Board D8-59, 2075 Bayview Avenue Toronto, ON M4N 3M5 Canada	
	203-25 Charlton Ave East Hamilton, ON L8N 1Y2 Canada	Institutional Review Board Services 372 Hollandview Trail, Suite 300 Aurora, ON L4G 0A5 Canada	
	Azienda Ospedaliera Universitaria Integrata Verona Policlinico G.B. Rossi, Instituto di Patologia Speciale Medica Cattedra di Reumatologia P. le L.A. Scuro, 10 Verona 37134 Italy	Comitato Etico per la sperimentazione clinica dei medicinali dell'Azienda Ospedaliera Universitaria Integrata Verona - P.le Stefani, 1 37126 Verona Italy	
	Clinical Pharmacology Study Group 25 Oak Avenue Worchester, MA 01605-0000 United States	Western International Review Board 3535 Seventh Avenue SW Olympia, WA 98502 United States	

List of Investigators and Corresponding IEC/IRB			
	**Site Name/Post Office Address**	**Name and Address of IEC/IRB**	
	Department of Rheumatology St. George Hospital Gray Street Kogarah, NSW 2217 Australia	South Eastern Sydney and Illawarra Health Human Research Ethics Committee St. George Hospital Gray Street Kogarah NSW 2217 Australia	
	Rheumatology Department Level 4, QEII Building Institute of Rheumatology & Orthopaedics Royal Prince Albert Hospital 59 Missenden Road Camperdown NSW 2050 Australia	Sydney Local Health District Ethics Review Committee (RPAH Zone) Royal Prince Alfred Hospital c/o Research Development Office Missenden Road Camperdown NSW 2050 Australia	

	List of Investigators and Corresponding IEC/IRB		
	**Site Name/Post Office Address**	**Name and Address of IEC/IRB**	
	Department of Rheumatology, Medical Centre Frederiksberg Hospital Nordre Fasanvej 57 2000 Frederiksberg Denmark	Den Videnskabsetiske Komité for Ringkjöbing, Ribe and Sönderjyllands Community Central Hospital Östergade 80 6700 Esbjerg Denmark	
	Hopital Avicenne Service de Rhumatologie 125, Rue de Stalingrad Bobigny 93009 France	CCPPRB de Paris St Louis – Hopital de St Louis 1 Avenue Claude Vellefaux Paris 75475 France	
	8263 Grove Avenue, Suite 100 Rancho Cucamonga, CA 91730 United States	Western International Review Board 3535 Seventh Avenue SW Olympia, WA 98502 United States	

	List of Investigators and Corresponding IEC/IRB		
	**Site Name/Post Office Address**	**Name and Address of IEC/IRB**	
	Fakultni Nemocnice Hradec Kralove II interni klinika Sokolska 581 Hradec Kralove 500 05 Czech Republic	Fakultni Nemocnice Hradec Kralove Eticka Komise Sokolska 581 Hradec Kralove 50005 Czech Republic	
	Karolinska University Hospital – Huddinge Department of Rheumatology Stockholm SE-141 86 Sweden	Regionala etikprovningsnamnden i Stockholm FE 289 (mail address) Karolinska Institutet Nobels vag 12A (visitors address) Stockholm 17177 Sweden	

List of Investigators and Corresponding IEC/IRB			
	Site Name/Post Office Address	Name and Address of IEC/IRB	
	Leiden University Medical Center Department of Rheumatology Albinusdreef 2 Leiden 2333 ZA The Netherlands	CWOM Academic Hospital Nijmegen Gerard van Swietenlaan 2 Nijmegen The Netherlands	

List of Investigators and Corresponding IEC/IRB			
	Site Name/Post Office Address	Name and Address of IEC/IRB	
	St. Vincent's University Hospital Department of Rheumatology The Bone and Joint Unit Elm Park Dublin 4 Ireland	Clinical Research Ethics Comm. of the Cork Teaching Hospitals Lancaster Hall 6 Little Hanover Street Cork Ireland	

List of Investigators and Corresponding IEC/IRB			
	Site Name/Post Office Address	Name and Address of IEC/IRB	
	Universitair Medisch Centrum Groningen<br>Afdeling Reumatologie Hanzeplein 1 Groningen 9713 GZ The Netherlands	Local: Commissie Mensgebonden Onderzoek (CMO)<br>Regio Arnhem-Nijmegen Radboud Universitair Medisch Centrum P.O. Box 9101 Geert Grooteplein 10 Nijmegen 6500 HB The Netherlands Central: Commissie Mensgebonden Onderzoek (CMO) Regio Arnhem-Nijmegen p/a UMC St. Radboud Huispost 531 Geert Grooteplein 10 Nijmegen 6525 GA The Netherlands	

List of Investigators and Corresponding IEC/IRB			
	**Site Name/Post Office Address**	**Name and Address of IEC/IRB**	
	Austin and Repatriation Medical Centre Repatriation Campus Rheumatology Department Banksia Street West Heidelberg VIC 3081 Australia	Austin Hospital<br>Human Research Ethics Committee Research Ethics Unit, Level 8, HSB - Room 8322 145 Studley Road Heidelberg VIC 3084 Australia	
	Charite Universitaetsmedizin Campus Virchow Klinikum Rheumatologie und Klinische Immunologie Chariteplatz 1 Berlin 10117 Germany	Local: Charite Universitaetsmedizin Campus Virchow Klinikum Ethikkommission Schumannstr. 20/21 Berlin 10117 Germany Central: Universitaet Erlangen-Nuernberg Ethik-Kommission der Medizinischen Fakultaet der Friedrich-Alexander-Universitaet Erlangen/Nuernberg Krankenhausstr. 12 Erlangen 91054 Germany	

List of Investigators and Corresponding IEC/IRB			
	**Site Name/Post Office Address**	**Name and Address of IEC/IRB**	
	Tampa Medical Group, PA 4700 North Habana Avenue Suite 303 Tampa, FL 33614 United States	Western International Review Board 3535 Seventh Avenue SW Olympia, WA 98502 United States	

List of Investigators and Corresponding IEC/IRB			
	**Site Name/Post Office Address**	**Name and Address of IEC/IRB**	
	Radiant Research, Gainesville<br>Gainesville Clinical Research Center<br>1014 NW 57th Street, Suite A, C & D<br>Gainesville, FL 32605 United States	Western International Review Board<br>3535 Seventh Avenue SW<br>Olympia, WA 98502 United States	
	Hospital Univ Clinic i Provincial<br>Servicio de Reumatologia 2 Piso,<br>Escalera 11, Despacho 13 C/<br>Villarroel 170 Barcelona 08036<br>Spain	Hospital Univ Clinic i Provincial<br>Comite Etico de Investigacion clinica C/<br>Villarroel, 170 Barcelona 08036 Spain	
	Arthritis Centre<br>800 Sherbrook Street, RR149<br>Winnipeg, MB R3A 1M4<br>Canada	University of Manitoba<br>Biomedical Research Ethics Board<br>A112-753 McDermot Avenue<br>Winnipeg, MB R3E 0W3<br>Canada	

List of Investigators and Corresponding IEC/IRB			
	**Site Name/Post Office Address**	**Name and Address of IEC/IRB**	
	Hospital Gregorio Maranon Doctor<br>Esquerdo, 46 28007 Madrid Spain	Comite Etico de Investigacion clinica<br>Hospital Gregorio Maranon Doctor<br>Esquerdo, 46 28007 Madrid Spain	

List of Investigators and Corresponding IEC/IRB			
	**Site Name/Post Office Address**	**Name and Address of IEC/IRB**	
	Centre Hospitalier Universitaire Vaudois<br>Service de Rhumatologie Hopital Orthopedique 4 Avenue Pierre Decker Lausanne 1011 Switzerland	Commission d'Ethique de la Recherche Clinique<br>21 rue du Bugnon Lausanne 1005 Switzerland	
	1301 West 38th Street Suite 609 Austin, TX 78705 United States	Western International Review Board 3535 Seventh Avenue SW Olympia, WA 98502 United States	
	Arthritis Center of Nebraska 3901 Pine Lake Road, Suite 120 Lincoln,NE 68516 United States	Western International Review Board 3535 Seventh Avenue SW Olympia, WA 98502 United States	

List of Investigators and Corresponding IEC/IRB			
	**Site Name/Post Office Address**	**Name and Address of IEC/IRB**	
	187 Hughson Street South Hamilton, ON L8N 2B6 Canada	Institutional Review Board Services 372 Hollandview Trail, Suite 300 Aurora, ON L4G 0A5 Canada	
	Metroplex Clinical Research Center 8144 Walnut Hill Lane, Suite 810 Dallas, TX 75231 United States	Western International Review Board 3535 Seventh Avenue SW Olympia, WA 98502 United States	

List of Investigators and Corresponding IEC/IRB			
	**Site Name/Post Office Address**	**Name and Address of IEC/IRB**	
	Hopital Lapeyronie Service d' Immuno - Rhumatologie 371, Avenue du Douen Gaston Giraud 34295 Montpellier Cedex 5 France	CCPPRB de Paris St Louis - Hopital St Louis Porte 5 du Carre historique – 1, avenue Claude Vellefaux 75475 Paris Cedex 10 France	
	Università di Genova Divisione de Reumatologia Dipartimento de Medicina Interna Viale Benedetto XV, 6 16132 Genova Italy	Comitato Etico del DIMI Università degli Studi di Genova Viale Benedetto XV, 6 16132 Genova Italy	
	Hjörring Hospital Department of Rheumatology Bispensgade 37 9800 Hjørring Denmark	Ethics committee for Ringkjøbing, Ribe and Sønderjylland The Secretariat Central hospital Østergade 80 DK 6700 Esbjerg Denmark	

List of Investigators and Corresponding IEC/IRB			
	**Site Name/Post Office Address**	**Name and Address of IEC/IRB**	
	Clinical Pharmacology and Toxicology Department Therapeutics Centre St. Vincent's Hospital Victoria Street Darlinghurst NSW 2010 Australia	St. Vincent’s Hospital Research Ethics Committee Level 6, de Lacy Building St. Vincent's Hospital 390 Victoria Street Darlinghurst NSW 2010 Australia	

List of Investigators and Corresponding IEC/IRB			
	**Site Name/Post Office Address**	**Name and Address of IEC/IRB**	
	AO Universita Santa Maria della Misericordia di Udine Clinica Medica Cattedra di Reumatologia Policlinico Universitario Piazza S. Maria Misericordia, 13 Udine 33100 Italy	Comitato Etico Aziendale dell'Azienda Ospedaliero Universitaria "Santa Maria della Misericordia" Via Colugna, 50 Udine 33100 Italy	

List of Investigators and Corresponding IEC/IRB			
	**Site Name/Post Office Address**	**Name and Address of IEC/IRB**	
	Cliniques Universitaires Saint Luc Department de Rhumatologie Avenue Hippocrate 10 Brussels 1200 Belgium	Comission d'Ethique Biomédical Hospitaux Facultaire Clinique Universitaire UCL Professeur Dr. Maloteaux Tour Harvey, UCL 5514 Avenue Hippocrate 10 1200 Bruxelles Belgium	
	Rockford Health Physicians 2300 N. Rockton Avenue Rockford, IL 61103 United States	Western International Review Board 3535 Seventh Avenue SW Olympia, WA 98502 United States	
	Coastal Clinical Research, Inc. 6701 Airport Blvd., Suite C-132 Mobile, AL 36608 United States	Western International Review Board 3535 Seventh Avenue SW Olympia, WA 98502 United States	

List of Investigators and Corresponding IEC/IRB			
	**Site Name/Post Office Address**	**Name and Address of IEC/IRB**	
	Rheumatologische Einheit der Ludwig-Maximilians-Universitaet Medizinische Poliklinik Pettenkoferstrasse 8a 80336 Muenchen Germany	Bayerische Landesaerztekammer Muehlbaurstr. 16 81677 Muenchen Germany	
	Groupe Hospitalier Cochin Service de Rhumatologie B Groupement universitaire hospitalier Ouest 27 rue du Faubourg St Jacques 75679 Paris Cedex 14 France	CCP IIe de France IV de Saint Louis Hopital Saint Louis 1 Avenue Claude Vellefaux Paris 75475 Cedex 10 France	

List of Investigators and Corresponding IEC/IRB			
	**Site Name/Post Office Address**	**Name and Address of IEC/IRB**	
	Centre Hospitalier Universitaire Vaudois Service de Rhumatologie Hopital Orthopedique 4 Avenue Pierre Decker Lausanne 1011 Switzerland	Commission d'Ethique de la Recherche Clinique 21 rue du Bugnon Lausanne 1005 Switzerland	
	Rheumatology Northwest PLLC and Clinical Trials Northwest 3902 Creekside Loop, Suite 120 Yakima, WA 98902 United States	Western International Review Board 3535 Seventh Avenue SW Olympia, WA 98502 United States	
	Arthritis Centre 800 Sherbooke Street, RR149 Winnkpeg, MB R3A 1M4 Canada	University of Manitoba Biomedical Research Ethics Board A112-753 McDermot Avenue Winnipeg, MB R3E 0W3 Canada	

	List of Investigators and Corresponding IEC/IRB		
	**Site Name/Post Office Address**	**Name and Address of IEC/IRB**	
	Rheumatology Research & Rehabilitation Unit University of Leeds 36 Clarendon Road Leeds LS1 3EX UK	Local Research Ethics Committee 6th Floor Wellcome Wing The General Infirmary at Leeds Leeds, LS1 3EX UK Northern and Yorkshire MREC John Snow House Durham University Science Park Durham DH1 3YG UK	

	List of Investigators and Corresponding IEC/IRB		
	**Site Name/Post Office Address**	**Name and Address of IEC/IRB**	
	Zentrum fuer Therapiestudien Deutscher Platz 5 Leipzig 04103 Germany	Local: Ethik - Kommission an der Medizinischen Fakultaet der Universitaet Leipzig Haertelstrasse 16-18 Leipzig 04107 Germany Central: Universitaet Erlangen-Nuernberg Ethik-Kommission der Medizinischen Fakultaet der Friedrich-Alexander-Universitaet Erlangen/Nuernberg Krankenhausstr. 12 Erlangen 91054 Germany	
	1901 S. Cedar #201<br>Tacoma, WA 98405 United States	Western International Review Board<br>3535 Seventh Avenue SW Olympia, WA 98502 United States	

List of Investigators and Corresponding IEC/IRB			
	**Site Name/Post Office Address**	**Name and Address of IEC/IRB**	
	Clinica Medica Cattedra di Reumatologia Policlino Universitario Piazza S. Maria Misericordia, 1 Udine 33100 Italy	Comitato Etico Indipendente del Policlinico Universitario di Udine Via Colugna, 50 33100 Udine Italy	

List of Investigators and Corresponding IEC/IRB			
	**Site Name/Post Office Address**	**Name and Address of IEC/IRB**	
	St. Vincent's University Hospital Department of Rheumatology The Bone and Joint Unit Elm Park Dublin 4 Ireland	Clinical Research Ethics Comm. of the Cork Teaching Hospitals Lancaster Hall 6 Little Hanover Street Cork Ireland	

List of Investigators and Corresponding IEC/IRB			
	**Site Name/Post Office Address**	**Name and Address of IEC/IRB**	
	Rikshospitalet Rev. poliklinik Sognsvannsvn.20 NO-0027 Oslo Norway	Regional komite for Medisinsk Forskningsetikk Postboks 1130 Blindern 0318 Oslo Norway	
	2845 Aventura Blvd., Suite 100 Aventura FL 33180 United States	Western International Review Board 3535 Seventh Avenue SW Olympia, WA 98502 United States	
	Limburgs Universitair Centrum Biomedical Research Institute Diepenbeek 3590 Belgium	Commissie Medische Ethiek Biomedical Research Institute Limburgs Universitair Centrum Diepenbeek 3590 Belgium	

List of Investigators and Corresponding IEC/IRB			
	**Site Name/Post Office Address**	**Name and Address of IEC/IRB**	
	Bluegrass Clinical Research, Inc.<br>5512 Bardstown Road, Suite 2<br>Louisville, KY 40291 United States	Western International Review Board<br>3535 Seventh Avenue SW<br>Olympia, WA 98502 United States	
	Hospital Clinico Universitario de Santiago Department Reumatologia Travasia a Choupana s/n Santiago de Compostela 15706 Spain	Comite Etico de Investigacion Clinica de Galicia Servicio Galego de Saude Edificio Administrativo San Lazaro, s/n Santiago de Compostela 15703 Spain	

List of Investigators and Corresponding IEC/IRB			
	**Site Name/Post Office Address**	**Name and Address of IEC/IRB**	
	Immanuel-Krankenhaus, Rheumaklinik<br>Klinik-und Heimbetriebsgessellschaft mbH<br>Karower Strasse 11 Berlin-Buch 13125 Germany	Local: Ethik-Kommission der Arztekammer Berlin<br>Friedrichstrasse 16 Berlin 10969 Germany<br>Central: Universitaet Erlangen-Nuernberg Ethik-Kommission der Medizinischen Fakultaet der Friedrich-Alexander-Universitaet Erlangen/Nuernberg Krankenhausstr. 12 Erlangen 91054 Germany	

	**List of Investigators and Corresponding IEC/IRB**		
	**Site Name/Post Office Address**	**Name and Address of IEC/IRB**	
	Klinikum der Universitaet Munich Rheumatologie/Klinische Studien Pettenkoferstrasse 8a Munich 80336 Germany	Universitaet Erlangen-Nuernberg Ethik-Kommission der Medizinischen Fakultaet der Friedrich-Alexander-Universitaet Erlangen/Nuernberg Krankenhausstr. 12 Erlangen 91054 Germany	

	**List of Investigators and Corresponding IEC/IRB**		
	**Site Name/Post Office Address**	**Name and Address of IEC/IRB**	
	Uppsala University Hospital Akademiska sjukhuset Department of Rheumatology Uppsala 75185 Sweden	Regionala etikprovningsnamnden i Stockholm FE 289 (mail address) Karolinska Institutet Nobels vag 12A (visitors address) Stockholm 17177 Sweden	
	Houston Institute for Clinical Research 7777 Southwest Freeway Suite 720 Houston, TX 77074 United States	Western International Review Board 3535 Seventh Avenue SW Olympia, WA 98502 United States	
	Garvan Institute Building 384 Victoria Street Darlinghurst NSW 2010 Australia	St. Vincent's Hospital Research Ethics Committee St. Vincent's Hospital Victoria Street Darlinghurst, NSW 2010 Australia	

	**List of Investigators and Corresponding IEC/IRB**		
	**Site Name/Post Office Address**	**Name and Address of IEC/IRB**	
	Suite 13 Surgicentre 38 Ranelagh Crescent South Perth, WA 6151 Australia	Sir Charles Gairdner Hospital Institutional Research Ethics Committee Department of Rheumatology C Block, 1st Floor, Room S1-88 Sir Charles Gairdner Hospital Verdun Street Nedlands, WA 6009 Australia	

	List of Investigators and Corresponding IEC/IRB		
	**Site Name/Post Office Address**	**Name and Address of IEC/IRB**	
	Institut de Rhumatologie de Montreal 1551 rue Ontario Est, 2e étage Montreal, QC H2L 1S6 Canada	Institutional Review Board Services 372 Hollandview Trail, Suite 300 Aurora, ON L4G 0A5 Canada	
	4004 Ben Franklin Boulevard Durham, NC 27704 United States	Western International Review Board 3535 Seventh Avenue SW Olympia, WA 98502 United States	
	Rheumatology Research Unit Level 6-Box 194 Addenbrookes Hospital Hills Road Cambridge CB2 QQ	Cambridge LREC Box 148 Addenbrookes NHS Trust Hills Road Cambridge CB2 2QQ	

	List of Investigators and Corresponding IEC/IRB		
	**Site Name/Post Office Address**	**Name and Address of IEC/IRB**	
	Medizinische Universitaetsklinik Graz Universitaetsklinik fuer Innere Medizin Klinische Abteilung fuer Rheumatologie Auenbruggerplatz 15 8036 Graz Austria	Ethikommission des Landeskrankenhauses Universitatskliniken Graz Auenbruggerplatz 29 8036 Graz Austria	
	Fundacion Jimenez Diaz Clinica Ntra Sra de la Concepcion Avda. de Reyes Catolicos, 2 28040 Madrid Spain	CEIC Fundacion Hospital Jiménez Díaz Clínica Ntra Sra de la Concepción Avda. de Reyes Católicos, 2 28040 Madrid Spain	
	Denver Arthritis Clinic 200 Spruce Street #100 Denver, CO 80230 Untied States	Western International Review Board 3535 Seventh Avenue SW Olympia, WA 98502 United States	

	List of Investigators and Corresponding IEC/IRB		
	**Site Name/Post Office Address**	**Name and Address of IEC/IRB**	
	Grasten Gigthospital Research Unit Nygade 29 6300 Grasten Denmark	Ethics Committee for Ringkjøbing, Ribe and Sønderjylland The Secretariat Centralhospital Østergade 80 DK 6700 Esbjerg Denmark	

	List of Investigators and Corresponding IEC/IRB		
	**Site Name/Post Office Address**	**Name and Address of IEC/IRB**	
	Arthritis Health<br>9097 E. Desert Cove, Suite 100<br>Scottsdale, AZ 85260 United States	Western International Review Board<br>3535 Seventh Avenue SW Olympia, WA<br>98502 United States	
	McBride Clinic 1110 N. Lee<br>Oklahoma City, OK 73103 United<br>States	Western International Review Board 3535<br>Seventh Avenue SW Olympia, WA 98502<br>United States	

	List of Investigators and Corresponding IEC/IRB		
	**Site Name/Post Office Address**	**Name and Address of IEC/IRB**	
	9850 Genessee Avenue, Suite 810<br>La Jolla, CA 92037<br>United States	Western International Review Board<br>3535 Seventh Avenue SW<br>Olympia, WA 98502 United States	
	Hobart Rheumatology Practice<br>4 Warneford Street South Hobart<br>TAS 7004 Australia	Southern Tasmania Health & Medical<br>Human Research Ethics Committee Research<br>& Development Office University of<br>Tasmania Private Bag 01 Hobart TAS 7001<br>Australia	
	Hospital Universitario Clinico San<br>Carlos Servicio de Reumatologia<br>Calle del Prof Martin Lagos, s/n<br>Madrid 28040 Spain	Hospital Universitario Germans Trias i Pujol<br>Comite Etico de Investigacion Clinica Ctra.<br>de Canyet, s/n Badalona - Barcelona 08915<br>Spain	

	List of Investigators and Corresponding IEC/IRB		
	**Site Name/Post Office Address**	**Name and Address of IEC/IRB**	
	1945 Versailes Street Suite 101<br>Ssarasota, FL 34239 United States	Western International Review Board 3535<br>Seventh Avenue SW Olympia, WA 98502<br>United States	

	List of Investigators and Corresponding IEC/IRB		
	**Site Name/Post Office Address**	**Name and Address of IEC/IRB**	
	Med. Klinik III mit Poliklinik der Friedrich-Alexander-Universitaet Rheumatologische Studienambulanz Krankenhausstrasse 12 91054 Erlangen Germany	Ethik-Kommission der Medizinischen Fakultaet der Friedrich-Alexander-Universitaet Erlangen-Nuernberg Universitaetsstrasse 40 91054 Erlangen Germany	
	Finnish Rheumatism Foundation Hospital Rheumatism Foundation Hospital Pikijaerventie 1 Heinola FI-18120 Finland	Hospital District of Helsinki and Uusimaa Ethics Committee Department of Medicine Box 340 Hus 00029 Finland	

	List of Investigators and Corresponding IEC/IRB		
	**Site Name/Post Office Address**	**Name and Address of IEC/IRB**	
	UCSD/Thornton Hospital<br>9320 Campus Point Drive<br>Suite A-225<br>La Jolla, CA 92037-0943<br>United States	UCSD Human Subjects Protection Program<br>La Jolla Village Professional Center<br>8950 La Jolla Village Drive<br>La Jolla, CA 92037-0052<br>United States	
	Fachkrankenhaus fuer Orthopaedie und Rheumatologie Klinikum fuer Rheumatologie Sophie von Boetticher Str. 1<br>Vogelsang-Gommern 39245 Germany	Local: Fachkrankenhaus fuer Orthopaedie und Rheumatologie Ethik-Kommission der Otto-von-Guericke-Universitaet an der medizinischen Fakultaet Leipziger Str. 44 Magdeburg 39120 Germany<br><br>Central: Universitaet Erlangen-Nuernberg Ethik-Kommission der Medizinischen Fakultaet der<br>Friedrich-Alexander-Universitaet Erlangen/Nuernberg Krankenhausstr. 12 Erlangen 91054 Germany	

	List of Investigators and Corresponding IEC/IRB		
	**Site Name/Post Office Address**	**Name and Address of IEC/IRB**	
	Rheumatologische Einheit der Ludwig-Maximilians-Universitaet Medizinische Poliklinik Pettenkoferstrasse 8a 80336 Munich Germany	Bayerische Landesaerztekammer Muehlbaurstr. 16 D-81677 Muechen Germany	
	105 West 8th Avenue Suite 6080 Spokane, WA 99204 United States	Western International Review Board 3535 Seventh Avenue SW Olympia, WA 98502 United States	

	List of Investigators and Corresponding IEC/IRB		
	**Site Name/Post Office Address**	**Name and Address of IEC/IRB**	
	The Division of Advanced Therapeutics Mount Sinai Hospital 60 Murray Street, 2nd Floor, Rm 205 Toronto, ON M5T 3L9 Canada	Research Ethics Board Mount Sinai Hospital 600 University Avenue, Rm 1003A Toronto, ON M5G 1X5 Canada	
	120 Stavanger Drive, Suite 102 St. John's, NL A1A 5E8 Canada	Human Research Ethics Board 95 Bonaventure Avenue, Suite 200 St. John's, NL A1B 2X5 Canada	
	Department of Rheumatology 4th Floor – Thomas Guy House Guy's Hospital St. Thomas Street London SE1 9RT UK	Guy's Research Ethics Committee Department of Nephrology & Transplantation 5th Floor – Thomas Guy House Guy's Hospital London SE1 9RT UK	

List of Investigators and Corresponding IEC/IRB			
	Site Name/Post Office Address	Name and Address of IEC/IRB	
	175 Meadowbrook Lane Duncansville, PA 16635 United States	Western International Review Board 3535 Seventh Avenue SW Olympia, WA 98502 United States	

List of Investigators and Corresponding IEC/IRB			
	Site Name/Post Office Address	Name and Address of IEC/IRB	
	Karolinska Hospital Dept. of Rheumatology 171 76 Stockholm Sweden	Karolinska Institute's Regional Research Ethics Committee Nobels väg 5 171 76 Stockholm Sweden	

List of Investigators and Corresponding IEC/IRB			
	Site Name/Post Office Address	Name and Address of IEC/IRB	
	Klein & Associates, MD, PA The Osteoporosis & Clinical Trials Center 346/348 Mill Street Hagerstown, MD 21740 United States Klein & Associates, MD, PA The Osteoporosis & Clinical Trials Center 921 Seton Drive, Suite C & D Cumberland, MD 21502 United States	Western International Review Board 3535 Seventh Avenue SW Olympia, WA 98502 United States	

List of Investigators and Corresponding IEC/IRB			
	**Site Name/Post Office Address**	**Name and Address of IEC/IRB**	
	Uppsala University Hospital Akademiska sjukhuset Department of Rheumatology Uppsala 75185 Sweden	Regionala etikprovningsnamnden i Stockholm FE 289 (mail address) Karolinska Institutet Nobels vag 12A (visitors address) Stockholm 17177 Sweden	
	Pacific Arthritis Center Medical Group 607 East Plaza Drive, Suite A Santa Maria, CA 93454 United States	Western International Review Board 3535 Seventh Avenue SW Olympia, WA 98502 United States	

List of Investigators and Corresponding IEC/IRB			
	**Site Name/Post Office Address**	**Name and Address of IEC/IRB**	
	Immanuel-Krankenhaus, Rheumaklinik<br>Klinik-und Heimbetriebsgessellschaft mbH Karower Strasse 11 Berlin-Buch 13125 Germany	Local: Ethik-Kommission der Arztekammer Berlin<br>Friedrichstrasse 16 Berlin 10969 Germany<br>Central: Universitaet Erlangen-Nuernberg Ethik-Kommission der Medizinischen Fakultaet der Friedrich-Alexander-Universitaet Erlangen/Nuernberg Krankenhausstr. 12 Erlangen 91054 Germany	
	The Center for Rheumatology<br>1367 Washington Ave., Suite 101<br>Albany, NY 12206 United States	Western International Review Board<br>3535 Seventh Avenue SW Olympia, WA 98502 United States	

List of Investigators and Corresponding IEC/IRB			
	**Site Name/Post Office Address**	**Name and Address of IEC/IRB**	
	Diakonihjemmets Sykehus Rev. poliklinik Boks 23 Vinderen NO-0319 Oslo Norway	Regional komite for Medisinsk Forskningsetikk Postbooks 1130 Blindern NO-0318 Oslo Norway	
	Arthritis Associates & Osteoporosis Center of Colorado Springs 215 Parkside Drive, Suite 200 Colorado Springs, CO 80910 United States	Western International Review Board 3535 Seventh Avenue SW Olympia, WA 98502 United States	
	Rheumazentrum Baden Baden Rotenbachtalstrasse 5 76530 Baden Baden Germany	Landesaerztekammer Baden-Wuerttemberg Koerperschaft des oeffentlichen Rechts Jahnstrasse 40 70597 Stuttgart Germany	

List of Investigators and Corresponding IEC/IRB			
	**Site Name/Post Office Address**	**Name and Address of IEC/IRB**	
	Goatcher Clinical Research Unit Department of Rheumatology C Block, 1st Floor, Room S1-88 Sir Charles Gairdner Hospital Verdun Street Nedlands, Western Australia 6009 Australia	Human Research Ethics Committee Sir Charles Gairdner Hospital Verdun Street Nedlands, WA 6009 Australia	
	Helsinki University Central Hospital Reumatologian klinikka Box 263 HUS Helsinki 00029 Finland	Hospital District of Helsinki and Uusimaa Ethics Committee Department of Medicine Box 340 Hus Helsinki 00029 Finland	

	List of Investigators and Corresponding IEC/IRB		
	**Site Name/Post Office Address**	**Name and Address of IEC/IRB**	
	Rheumatology Department 3rd Floor, E Block Monash Medical Centre 246 Clayton Road Clayton VIC 3168 Australia	Southern Health Human Research Ethics Committee Monash Medical Centre 246 Clayton Road Clayton VIC 3168 Australia	

	List of Investigators and Corresponding IEC/IRB		
	**Site Name/Post Office Address**	**Name and Address of IEC/IRB**	
	East Penn Rheumatology Associates, PC 701 Ostrum Street, Suite 402 Bethlehem, PA 18015 United States	Western International Review Board 3535 Seventh Avenue SW Olympia, WA 98502 United States	

	List of Investigators and Corresponding IEC/IRB		
	**Site Name/Post Office Address**	**Name and Address of IEC/IRB**	
	Univ. Klinik fuer Innere Medizin III, Klin. Abt. fuer Rheumatologie Waehringer Guertel 18-20 1090 Vienna Austria	Ethik-Kommission der Medizinischen Universitaet Wien und des Allgemeinen Krankenhauses der Stadt Wien AKH Borschkegasse 8b/6 1090 Vienna Austria	
	Research Point 1594 Route 9 Bldg. 1 Toms River, NJ 08755 United States	Western International Review Board 3535 Seventh Avenue SW Olympia, WA 98502 United States	
	Department of Rheumatology Ysbyty Gwynedd Hospital Penrhosgarnedd Bangor LL57 2PW UK	Clinical Governance Support Unit Ysbyty Gwynedd Bangor LL57 2PW UK	
	PRO Research 633 E. 11th Avenue Eugene, OR 97401 United States	Western International Review Board 3535 Seventh Avenue SW Olympia, WA 98502 United States	

	List of Investigators and Corresponding IEC/IRB		
	**Site Name/Post Office Address**	**Name and Address of IEC/IRB**	
	Rheumatology Department<br>Medical Outpatient Department<br>Level 2 North Block The Royal<br>Newcastle Center Lookout Road<br>New Lambton NSW 2305 Australia	Hunter New England Research Ethics Unit<br>Locked Bag 1, Lookout Road New Lambton<br>NSW 2305 Australia	

	List of Investigators and Corresponding IEC/IRB		
	**Site Name/Post Office Address**	**Name and Address of IEC/IRB**	
	CHU Sart Tilman, Liege<br>Domaine Universitaire du Sart<br>Tilman Batiment B35 Liege 4000<br>Belgium	CHU Sart Tilman, Liege<br>Faculty of Medicine, Ethics Committee<br>University of Liege Domaine Universitaire<br>Sart-Tilman Liege 4000 Belgium	
	346 Mill Street<br>Hagerstown, MD 21740<br>United States	Western International Review Board<br>3535 Seventh Avenue SW<br>Olympia, WA 98502 United States	

List of Investigators and Corresponding IEC/IRB			
	**Site Name/Post Office Address**	**Name and Address of IEC/IRB**	
	Hospital Universitario Nuestra Senora de Valme Ctra. Cadiz, s/n Sevilla 41014 Spain	Comite Etico de Investigacion Clinica Hospital Universitario de Valme Ctra. Cadiz, s/n 41014 Sevilla Spain	
	Hospital General de Alicante<br>Pintor Baeza, s/n<br>03010 Alicante Spain	Comité Etico de Investigaión Clínicínic<br>Hospital General Alicante<br>Pinto Baeza, s/n 03010 Alicante Spain	
	Oddeleni klinicke farmakologie<br>Fakultni nemocnice Plzen<br>Dr. E. Benese 13<br>Plzen 305 99<br>Czech Republic	Eticka Komise FN a LF UK v Plzni<br>Dr. E. Benese 13<br>Plzen 305 99<br>Czech Republic	
	Rheumazentrum Baden Baden<br>Rotenbachtalstrasse 5<br>76530 Baden Baden Germany	Landesaerztekammer Baden-Wuerttemberg<br>Koerperschaft des oeffentlichen Rechts<br>Jahnstrasse 40 70597 Stuttgart Germany	

List of Investigators and Corresponding IEC/IRB			
	**Site Name/Post Office Address**	**Name and Address of IEC/IRB**	
	Cork University Hospital Department of Rheumatology Wilton Road Cork Ireland	Clinical Res. Ethics Comm. of the Cork Teaching Hospitals Lancaster Hall 6 Little Hanover Street Cork Ireland	
	Arthritis Care Center<br>1835 Park Avenue<br>San Jose, CA 95126 United States	Western International Review Board<br>3535 Seventh Avenue SW<br>Olympia, WA 98502 United States	

	List of Investigators and Corresponding IEC/IRB		
	**Site Name/Post Office Address**	**Name and Address of IEC/IRB**	
	Rheumatology Research Unit Sunshine Coast Maroochy Waters Shopping Centre 9-10 Denna Street Maroochydore QLD 4558 Australia	Royal Brisbane & Women's Hospital Human Research Ethics Committee Level 7, Block 7 Royal Brisbane & Woman's Hospital Butterfield Street Herston QLD 4029 Australia	
	Groupe Hospitalier Cochin<br>Service de Rhumatologie B Groupement universitaire hospitalier Ouest 27 rue du Faubourg St Jacques 75679 Paris Cedex 14 France	CCP IIe de France IV de Saint Louis Hopital Saint Louis<br>1 Avenue Claude Vellefaux Paris 75475 Cedex 10 France	

	List of Investigators and Corresponding IEC/IRB		
	**Site Name/Post Office Address**	**Name and Address of IEC/IRB**	
	Oulu University Hospital<br>P.O. Box 20<br>FIN-90029 OYS Finland	Hospital District of Helsinki and Uusimaa Ethics Committee Department of Medicine PO Box 340 FIN-00020 HUS Finland	
	Centrallasarettet<br>Reumatologkliniken<br>721 89 Västaras Sweden	Karolinska Instituts Regional Research Ethics Committee<br>Nobels väg 5 171 76 Stockholm Sweden	

	List of Investigators and Corresponding IEC/IRB		
	**Site Name/Post Office Address**	**Name and Address of IEC/IRB**	
	Hospital General de Alicante Servicio de Reumatologia C/. Maestro Alonso s/n Alicante 03010 Spain	CEIC Hospital General Universitario de Alicante Planta 3. Edificio gris C/. Maestro Alonso 109 Alicante 03010 Spain	
	Revmatologicky ustav<br>Na Slupi 4 Prague 2 128 50 Czech Republic	Eticka komise Revmatologicky ustav<br>Na Slupi 4 Prague 2 128 50 Czech Republic	
	CHU Rennes - Hospital Sud<br>Service de Rhumatologie 16 Boulevard de Bulgarie Rennes 35056 France	CCPPRB de Paris St Louis - Hopital St Louis Porte 5 du Carre historique – 1, avenue Claude Vellefaux 75475 Paris Cedex 10 France	

	List of Investigators and Corresponding IEC/IRB		
	**Site Name/Post Office Address**	**Name and Address of IEC/IRB**	
	Klinikum der Albert-Ludwigs-Universitaet Abteilung IV Rheumatologie Hugstetter Strasse 55 Freiburg 79106 Germany	Local: Klinikum der Albert-Ludwigs-Universitaet Ethik-Kommission Engelberger Str. 21 Freiburg 79106 Germany Central: Universitaet Erlangen-Nuernberg Ethik-Kommission der Medizinischen Fakultaet der Friedrich-Alexander-Universitaet Erlangen/Nuernberg Krankenhausstr. 12 Erlangen 91054 Germany	
	1301 West 38th Street<br>Suite 110<br>Austin, TX 78705 United States	Western International Review Board<br>3535 Seventh Avenue SW<br>Olympia, WA 98502 United States	

List of Investigators and Corresponding IEC/IRB			
	**Site Name/Post Office Address**	**Name and Address of IEC/IRB**	
	Rheumatology Department Queen Elizabeth Hospital Woodville Road Woodville SA 5011 Australia	North Western Adelaide Health Service Ethics of Human Research Queen Elizabeth Hospital Woodville Road Woodville SA 5011 Australia	

List of Investigators and Corresponding IEC/IRB			
	**Site Name/Post Office Address**	**Name and Address of IEC/IRB**	
	Universitair Medisch Centrum Groningen<br>Dept Of Reumatology<br>Hanzeplein 1 Groningen 9713 GZ The Netherlands	Local: Commissie Mensgebonden Onderzoek (CMO)<br>Regio Arnhem-Nijmegen Radboud Universitair Medisch Centrum P.O. Box 9101 Geert Grooteplein 10 Nijmegen 6500 HB The Netherlands Central: Commissie Mensgebonden Onderzoek (CMO) Regio Arnhem-Nijmegen P/A UMC St. Radboud Huispost 531, Geert Grooteplein 10 Nijmegen 6525 GA The Netherlands	
	Health Core, Inc.<br>4735 Ogletown-Stanton Road, Suite 3201 Newark, DE 19713-2094 United States	Western International Review Board<br>3535 Seventh Avenue SW<br>Olympia, WA 98502 United States	

List of Investigators and Corresponding IEC/IRB			
	**Site Name/Post Office Address**	**Name and Address of IEC/IRB**	
	Norrlands Universitets sjukhus Rematologkliniken 901 85 Umea Sweden	Karolinska Institute's Regional Research Ethics Committee Nobels väg 5 171 77 Stockholm Sweden	
	Evangelisches Fachkrankenhaus Rheumatologie Rosenstrasse 2 40882 Ratingen Germany	Ethikkommission der Medizinischen Fakultaet der Heinrich Heine Universitaet Duesseldorf Moorenstrasse 5 40225 Duesseldorf Germany	
	12395 El Camino Real<br>Suite 117<br>San Diego, CA 92130 United States	Western International Review Board<br>3535 Seventh Avenue SW<br>Olympia, WA 98502 United States	

List of Investigators and Corresponding IEC/IRB			
	**Site Name/Post Office Address**	**Name and Address of IEC/IRB**	
	Rheumatology Department Level 5C, Tower Block Queen Elizabeth Hospital Woodville Road Woodville SA 5011 Australia	Central Northern Adelaide Health Service Ethics of Human Research Committee South Eastern Sydney and Illawarra Health Queen Elizabeth Hospital Woodville Road Woodville SA 5011 Australia	

List of Investigators and Corresponding IEC/IRB			
	**Site Name/Post Office Address**	**Name and Address of IEC/IRB**	
	Vasteras Hospital<br>Department of Rheumatology<br>Vasteras S-72189 Sweden	Regionala etikprovningsnamnden i Stockholm<br>FE 289 (mail address) Karolinska Institutet Nobels vag 12A (visitors address) Stockholm 17177 Sweden	
	Goatcher Clinical Research Unit<br>Royal Perth Hospital<br>Shenton Park Campus Thorburn House, 6 Selby Street Shenton Park WA 6008 Australia	Royal Perth Hospital Ethics Committee<br>Kirkman House<br>Wellington Street Campus GPO Box X2213 Perth WA 6847 Australia	
	Narodny ustav reumatickych chorob<br>Nabrezie Ivana Krasku 4 Piestany 92112 Slovakia	Eticka komisia pri Nurch<br>Narodnem ustavu reumatickych chorob<br>Nabrezie Ivana Krasku 4 Piestany 321 01 Slovakia	

List of Investigators and Corresponding IEC/IRB			
	**Site Name/Post Office Address**	**Name and Address of IEC/IRB**	
	562 Heritage Medical Research Centre University of Alberta Edmonton, AB T6G 2S2 Canada	University of Alberta Health Research Ethics Board 308 Campus Tower University of Alberta Edmonton, AB T6G 1K8 Canada	
	Hopital Lapeyronie Service d' Immuno - Rhumatologie 371, Avenue du Douen Gaston Giraud 34295 Montpellier Cedex 5 France	CCPPRB de Paris St Louis - Hopital Saint Louis Porte 5 du Carre historique – 1, avenue Claude Vellefaux 75475 Paris Cedex 10 France	
	Austin Rheumatology Research<br>1301 West 38th Street, Suite 110<br>Austin, TX 78705 United States	Western International Review Board<br>3535 Seventh Avenue SW<br>Olympia, WA 98502 United States	

List of Investigators and Corresponding IEC/IRB			
	**Site Name/Post Office Address**	**Name and Address of IEC/IRB**	
	Universitaet Muenchen Rheumaeinheit Pettenkoferstr. 8a D-80336 Muenchen Germany	Bayrische Landesaerztekammer Muenebaurstrasse 16 D-81677 Muenchen Germany	
	6525 West Sack Drive Suite 108 Glendale, AZ 85308 United States	Western International Review Board 3535 Seventh Avenue SW Olympia, WA 98502 United States	

List of Investigators and Corresponding IEC/IRB			
	**Site Name/Post Office Address**	**Name and Address of IEC/IRB**	
	Universitaet Erlangen-Nuernberg Med. Klinik III mit Poliklinik Krankenhausstr. 12 Erlangen 91054 Germany	Universitaet Erlangen-Nuernberg Ethik-Kommission der Medizinischen Fakultaet der Friedrich-Alexander-Universitaet Erlangen/Nuernberg Krankenhausstr. 12 Erlangen 91054 Germany	

	List of Investigators and Corresponding IEC/IRB		
	**Site Name/Post Office Address**	**Name and Address of IEC/IRB**	
	Klinikum der Universitaet Munich Rheumatologie/Klinische Studien Pettenkoferstrasse 8a Munich 80336 Germany	Universitaet Erlangen-Nuernberg Ethik-Kommission der Medizinischen Fakultaet der Friedrich-Alexander-Universitaet Erlangen/Nuernberg Krankenhausstr. 12 Erlangen 91054 Germany	

	List of Investigators and Corresponding IEC/IRB		
	**Site Name/Post Office Address**	**Name and Address of IEC/IRB**	
	Denver Arthritis Clinic 200 Spruce Street, #100 Denver, CO 80230 United States	Western International Review Board 3535 Seventh Avenue SW Olympia, WA 98502 United States	
	Academisch Ziekenhuis Maastricht Rheumatology Department Debyelaan 25 Maastricht 6229 HX The Netherlands	Local: Commissie Wetenschappelijk Huispost 9101 Postbus 722 Nijmegen 6500 HB The Netherlands Central: Commissie Mensgebonden Onderzoek (CMO) Regio Arnhem-Nijmegen p/a UMC St. Radboud Huispost 531 Geert Grooteplein 10 Nijmegen 6525 GA The Netherlands	

List of Investigators and Corresponding IEC/IRB			
	**Site Name/Post Office Address**	**Name and Address of IEC/IRB**	
	Reuma center Sabbatsbergs Sjukhus Olivekronasvag 5 Stockholm 11324 Sweden	Regionala etikprovningsnamnden i Stockholm FE 289 (mail address) Karolinska Institutet Nobels vag 12A (visitors address) Stockholm 17177 Sweden	
	CRIA Research 5333 N. Dixie Highway Suite 110 Fort Lauderdale, FL 33334 United States	Western International Review Board 3535 Seventh Avenue SW Olympia, WA 98502 United States	
	c/o Bioclin Health Care, Inc. 6091 Gilbert Road, Suite 520 Richmond, BC V7C 5L9 Canada	Institutional Review Board Services 372 Hollandview Trail, Suite 300 Aurora, ON L4G 0A5 Canada	

List of Investigators and Corresponding IEC/IRB			
	**Site Name/Post Office Address**	**Name and Address of IEC/IRB**	
	Hautepierre Hospital<br>Service de Rhumatologie CHU de Strasbourg 1, Avenue Moliere 67098 Strasbourg, Cedex 1 France	CCP IIe de France IV de Saint Louis Hopital Saint Louis 1 Avenue Claude Vellefaux 75475 Paris Cedex 10 France	
	Florida Medical Clinic, PA<br>Clinical Research Division 38135 Market Square Zephyrhillis, FL 33542 United States	Western International Review Board<br>3535 Seventh Avenue SW Olympia, WA 98502 United States	

	List of Investigators and Corresponding IEC/IRB		
	**Site Name/Post Office Address**	**Name and Address of IEC/IRB**	
	Allgem. Krankenhaus der Stadt Wien<br>Klinik f. Innere Medizin III/Rheumatologie Wahringer Gurtel 18-20 Vienna 1090 Austria	Ethikkommission der Medizinischen Universität Wien und des Allgemeinen Krankenhauses der Stadt Wien – AKH Borschkegasse 8b/E6 A-1090 Vienna Austria	
	Arthritis, Rheumatic & Back Disease Associates, PA<br>2309 Evesham Road, Suite 101<br>Voorhees, NJ 08043<br>United States	Western International Review Board<br>3535 Seventh Avenue SW<br>Olympia, WA 98502<br>United States	
	1144 Sonoma Avenue, Suite 101 Santa Rosa, CA 95405 United States	Western International Review Board 3535 Seventh Avenue SW Olympia, WA 98502 United States	

	List of Investigators and Corresponding IEC/IRB		
	**Site Name/Post Office Address**	**Name and Address of IEC/IRB**	
	Zentrum fuer Therapiestudien Wilhelm-Leuschner-Platz 12 04107 Leipzig Germany	Ethikkommission der Universitaet Leipzig Haertelstr. 16-18 04107 Leipzig Germany	
	West Island Rheumatology Research Associates 269 St. Jean Boulevard, Suite #209 Pointe Claire, QC H9R 3J1 Canada	Institutional Review Board Services<br>372 Hollandview Trail, Suite 300 Aurora, ON L4G 0A5 Canada	
	Rheumatology Department St. George Hospital Gray Street Kogarah NSW 2217 Australia	South Eastern Sydney Local Health Network Human Research Ethics Committee St. George Hospital Gray Street Kogarah NSW 2217<br>Australia	

List of Investigators and Corresponding IEC/IRB			
	**Site Name/Post Office Address**	**Name and Address of IEC/IRB**	
	Clinpharm International GmbH & Co. KG<br>Konsulplatz 3 Goerlitz 02826 Germany	Local: Ethik - Kommission an der Medizinischen<br>Fakultaet der Universitaet Leipzig<br>Haertelstrasse 16-18 Leipzig 04107 Germany<br><br>Central: Universitaet Erlangen-Nuernberg Ethik-Kommission der Medizinischen Fakultaet der<br>Friedrich-Alexander-Universitaet Erlangen/Nuernberg Krankenhausstr. 12 Erlangen 91054 Germany	
	Rheumatic Disease Center of Montreal 4060 Ste, Catherine St., West Suite 740 Montreal, QC H3Z 2Z3 Canada	Institutional Review Board Services<br>372 Hollandview Trail, Suite 300 Aurora, ON L4G 0A5 Canada	

List of Investigators and Corresponding IEC/IRB			
	**Site Name/Post Office Address**	**Name and Address of IEC/IRB**	
	Goatcher Clinical Research Unit<br>Royal Perth Hospital<br>Shenton Park Campus<br>Thorburn House, Selby Street<br>Shenton Park WA 6008 Australia	Royal Perth Hospital Ethics Committee<br>Royal Perth Hospital, Wellington Street Campus<br>GPO Box X2213 Perth WA 6847 Australia	

List of Investigators and Corresponding IEC/IRB			
	**Site Name/Post Office Address**	**Name and Address of IEC/IRB**	
	Centre Hospitalier Lyon Sud<br>Service de Rhumatologie Pav 2B<br>Chemin du Grand Revoyet Pierre Benite 69310 France	CCPPRB de Paris St Louis - Hopital Saint Louis Porte 5 du Carre historique – 1, avenue Claude Vellefaux 75475 Paris Cedex 10 France	

List of Investigators and Corresponding IEC/IRB			
	**Site Name/Post Office Address**	**Name and Address of IEC/IRB**	
	Hospital Universitario Germans Trias i Pujol Servicio de Reumatologia Crta. Del Canyet s/n Barcelona 08915 Spain	Comite Etico de Investigacion Clinica Hosp. Germans Trias i Pujol Ctra de Canyet s/n Barcelona 08915 Spain	
	CIADS Research Co., Ltd.<br>1835 Corydon Ave. Winnipeg, MB R3N 0K6 Canada	Institutional Review Board Services<br>372 Hollandview Trail, Suite 300 Aurora, ON L4G 0A5 Canada	

List of Investigators and Corresponding IEC/IRB			
	**Site Name/Post Office Address**	**Name and Address of IEC/IRB**	
	The Arthritis Program Research Group, Inc. 43 Lundy's Lane Newmarket, ON L3Y 3R7 Canada	Institutional Review Board Services<br>372 Hollandview Trail, Suite 300 Aurora ON L4G 0A5 Canada	
	6640 SW Redwood Lane<br>Suite 301<br>Portland, OR 97224 United States	Western International Review Board<br>3535 Seventh Avenue SW<br>Olympia, WA 98502 United States	
	Universita di Padova<br>Cattedra e Divisione di Reumatologia Policlinico Via Giustiniani 2 35128 Padova Italy	Comitato Etico per la Sperimentazione dell'Azienda Ospedaliera di Padova Via Giustiniani 2 35128 Padova Italy	

List of Investigators and Corresponding IEC/IRB			
	**Site Name/Post Office Address**	**Name and Address of IEC/IRB**	
	Hospital Universitario de Guadalajara Servicio de Reumatologia Donantes de Sangre s/n Guadalajara 19002 Spain	Comite Etico de Investigacion Clinica Hospital Universitario Germans Trias i Pujol Ctra. de Canyet, s/n Badalona - Barcelona 08916 Spain	
	Arthritis, Osteoporosis & Musculoskeletal Disease Center 280 Pleasant Street Concord, NH 03301 United States	Concord Hospital Human Investigation Committee Concord Hospital 250 Pleasant Street Concord, NH 03301 United States	
	#307, 4256 Bathurst Street Downsview, ON M3H 5Y8 Canada	IRB Services 14745-6 Yonge Street, Suite 328 Aurora, ON L4G 6H8 Canada	
	Arthritis Specialist of Western Michigan 1900 Wealthy SE, Suite 220 Grand Rapids, MI 49506 United States	Western International Review Board 3535 Seventh Avenue SW Olympia, WA 98502 United States	

List of Investigators and Corresponding IEC/IRB			
	**Site Name/Post Office Address**	**Name and Address of IEC/IRB**	
	AO Universitaria Seconda Universita degli Studi di Napoli Cattedra di Reumatologia Policlinico II Via Pansini 5 Naples 80131 Italy	Comitato Etico dell'Azienda Ospedaliera Universitaria della Seconda Universita degli studi di Napoli Via Costantinopoli 104 80138 Napoli Italy	
	U.Z. Ghent Universitair Ziekenhuis / University Hospital Afdeling Reumatologie / Department of Rheumatology De Pintelaan 185 Ghent 9000 Belgium	Commissie Medische Ethiek U.Z. Ghent De Pintelaan 185 9000 Gent Belgium	

List of Investigators and Corresponding IEC/IRB			
	**Site Name/Post Office Address**	**Name and Address of IEC/IRB**	
	Academic Hospital Maastricht Dept. of Rheumatology P. Debeyelaan 25 6229 HX Maastricht The Netherlands	CWOM Academic Hospital Nijmegen Gerard van Swietenlaan 2 Nijmegen The Netherlands 6500 HB Nijmegen The Netherlands	
	Academic Hospital Groningen Dept. of Rheumatology Hanzeplein 1 9713 GZ Groningen The Netherlands	Commissie Mensgebonden Onderzoek Regio Arnhem-Nijmegen UMC St Radboud Geert Grooteplein 10 Postbus 9101 6500 NB Nijmegen The Netherlands	

List of Investigators and Corresponding IEC/IRB			
	**Site Name/Post Office Address**	**Name and Address of IEC/IRB**	
	Universitair Medisch Centrum (UMC) St Radbourd Afdeling Reumatologie Geert Grooteplein 8 6525 GA Nijmegen, The Netherlands	Commissie Mensgebonden Onderzoek Regio Arnhem-Nijmegen UMC St Radboud Geert Grooteplein 10 Postbus 9101 6500 NB Nijmegen The Netherlands	
	Academic Hospital Groningen Dept. of Rheumatology Hanzeplein 1 9713 GZ Groningen The Netherlands	CWOM Academic Hospital Nijmegen Gerard van Swietenlaan 2 Nijmegen The Netherlands	
	Karolinska Hospital Dept. of Rheumatology 171 76 Stockholm Sweden	Karolinska Institute's Regional Research Ethics Committee Nobels väg 5 171 76 Stockholm Sweden	

List of Investigators and Corresponding IEC/IRB			
	**Site Name/Post Office Address**	**Name and Address of IEC/IRB**	
	Rheumatology Department<br>5th Floor, Building 1 Princess Alexandra Hospital Ipswich Road Woolloongabba QLD 4102 Australia	Human Research Ethics Committee<br>Level 2, Building 35 Princess Alexandra Hospital Ipswich Road Woolloongabba QLD 4102 Australia	
	UZ VUB Universitair Ziekenhuis Vrije Universiteit Brussels Rheumatology Unit Laarbeeklaan 101 Brussels 1090 Belgium	Commissie Medische Ethiek<br>UZ Brussels Faculteit Geneeskunde en Farmacie Laarbeeklaan 101 Brussels 1090 Belgium	
	U.Z. Ghent Universitair Ziekenhuis / University Hospital Afdeling Reumatologie / Department of Rheumatology De Pintelaan 185 Ghent 9000 Belgium	Ethics Committee University Hospital De Pintelaan 185 9000 Gent Belgium	

List of Investigators and Corresponding IEC/IRB			
	**Site Name/Post Office Address**	**Name and Address of IEC/IRB**	
	Hospital Universitario de Guadalajara Servicio de Reumatologia Donantes de Sangre s/n Guadalajara 19002 Spain	Hospital Universitario Germans Trias i Pujol Comite Etico de Investigacion Clinica Ctra. de Canyet, s/n Badalona - Barcelona 08915 Spain	
	Consulmed GbR<br>Konsulplatz 3<br>02826 Goerlitz Germany	Ethikkommission der Universitaet Leipzig<br>Haertelstrasse 16-18<br>04107 Leipzig Germany	
	Musculoskeletal Unit<br>Freeman Hospital Newcastle – upon Tyne NE7 7DN Great Britain	Prof. Heasman<br>Joint Ethics Committee Newcastle & North Tynesise Health Authority Benfeild Road Newcastle NE6 4PF Great Britain	

	List of Investigators and Corresponding IEC/IRB		
	**Site Name/Post Office Address**	**Name and Address of IEC/IRB**	
	Evangelisches Fachkrankenhaus Studienambulanz Dr. Wassenberg Rosenstrasse 2 Ratingen 40882 Germany	Local: Ethik-Kommission der Aerztekammer Nordrhein Tersteegenstrasse 9 Duesseldorf 40474 Germany Central: Universitaet Erlangen-Nuernberg Ethik-Kommission der Medizinischen Fakultaet der Friedrich-Alexander-Universitaet Erlangen/Nuernberg Krankenhausstr. 12 Erlangen 91054 Germany	
	Arthritis Center of Nebraska 2121 South 56th Street Lincoln NE 68506 United States	Western International Review Board 3535 Seventh Avenue SW Olympia, WA 98502 United States	

	List of Investigators and Corresponding IEC/IRB		
	**Site Name/Post Office Address**	**Name and Address of IEC/IRB**	
	8700 Beverly Blvd., Suite B-131 Los Angeles, CA 90048 United States	Cedars-Sinai Medical Center IRB 8383 Wilshire Blvd. Los Angeles, CA 90048 United States	
	Houston Institute for Clinical Research 7777 Southwest Freeway Suite 720 Houston, TX 77074 United States	Western International Review Board 3535 Seventh Avenue SW Olympia, WA 98502 United States	
	UZ Gasthuisberg Herestraat 49 3000 Leuven Belgium	Etische Commissie Klinishche Studies Universitair Ziekenhuis Gasthuisberg Herestraat 49 3000 Leuven Belgium	

List of Investigators and Corresponding IEC/IRB			
	**Site Name/Post Office Address**	**Name and Address of IEC/IRB**	
	Department of Rheumatology Hereford County Hospital Hereford HR1 2ER UK	Herefordshare Districe Ethics Committee Victoria House Eign Street Hereford HR4 0AN UK	
	STAT Research, Inc. One Elizabeth Place West Medical Plaza Suite 230 Dayton, OH 45417 United States	Western International Review Board 3535 Seventh Avenue SW Olympia, WA 98502 United States	

List of Investigators and Corresponding IEC/IRB			
	**Site Name/Post Office Address**	**Name and Address of IEC/IRB**	
	Chesapeake Medical Research 5601 Loch Raven Blvd. Russell Morgan Bldg., Suite 510 Baltimore, MD 21239 United States	Western International Review Board 3535 Seventh Avenue SW Olympia, WA 98502 United States	
